# The effect of environmental regulations on innovation in heavy-polluting and resource-based enterprises: Quasi-natural experimental evidence from China

**DOI:** 10.1371/journal.pone.0239549

**Published:** 2020-12-03

**Authors:** Ting Liu, Meijuan Liu, Xiaohan Hu, Bangsheng Xie

**Affiliations:** School of Management, Fujian A&F University, Fuzhou, China; Institute for Advanced Sustainability Studies, GERMANY

## Abstract

Environmental protection regulations adopted by governments affect the microeconomic behavior of enterprises. The Chinese government began piloting the outgoing leading officials’ accountability audit of natural resources assets (OANRA) in some regions in 2014. Based on this quasi-natural experimental setting, this paper chose heavy-polluting and resource-based enterprises in pilot regions of China from 2011 to 2016 as examples for studying the impact of the OANRA on enterprise innovation and further examines the role of government subsidies in this process. The study finds that the OANRA has no significant impact on enterprise innovation. However, with support from government subsidies, the OANRA dramatically accelerates enterprise innovation investment. The results are still seen after applying propensity matching analysis (PSM), balancing panel data and deleting special provinces. Further analysis shows that this effect is more obvious among small-scale, state-owned enterprises that are located in areas with high degrees of marketization and high bank credit constraints. This study advances the research of the OANRA’s effects on the microeconomic behavior of enterprises. Moreover, the adjustment effect of government subsidies also provides great reference value to making rational use of policy to cooperate with the OANRA.

## 1. Introduction

With the rapid growth of China's economy since the reform and opening, environmental pollution and ecological imbalance problems have become increasingly serious. The Chinese government has great concern for environmental governance and ecological protection and has committed to sustainable economic and social development. In 2013, the Chinese government proposed establishing a balance sheet of natural resources, the OANRA practice, and built a lifelong audit system for ecological environmental destruction to accelerate the transformation and upgrading of the national economy and achieve the goals of sustainable development. Under the leadership of the China National Audit Office from 2014, more than 10 provinces and cities, such as Shandong, Hubei, Inner Mongolia, Hunan, Guizhou, Jiangsu, Guangxi, Fujian, Shaanxi, and Sichuan, have been piloted with the OANRA. The purpose of the OANRA is to implement leadership responsibilities in sustainable natural resource utilization and ecological environmental protection, to strengthen the accountability for environmental protection responsibilities of leading officials, and to improve the establishment of correct political performance values of the leading cadre so that the leading cadre will not blindly pursue regional GDP growth or ignore the sustainable use of natural resources [1]. As a developing country with an emerging economy, China should not only take economic construction as its first priority but also pay attention to strengthening environmental protection. The promotion of technological innovation is an important way to adhere to the strategy of sustainable development and to harmonize economic construction with environmental protection. The enterprise is the basic unit of the microeconomy. It is the main body of environmental pollution and natural resource consumption but is also the main piece of national innovative behavior. Therefore, enterprise innovation is the key factor to achieving a win-win situation for environmental protection and economic growth. Thus, the promotion of technological innovation by enterprises becomes the key solution for the sustainable development of China’s economy.

Many studies have shown that local leader cadres in China have strong impacts on enterprise behavior in the transitioning economy [1–3]. The OANRA assesses local natural resource asset protection and economic growth and whether local leading officials will guide enterprises to conduct technological innovations to obtain sustainable growth of the local economy and protect natural resources under OANRA pressure. How does the innovative behavior of heavy-polluting and resource-based enterprises respond to changes in environmental regulations in the pilot areas? The answer to this question is relevant to the success of the OANRA. The existing research on the OANRA is mainly at the theoretical level. This article intends to use the OANRA pilots from 2014 as a quasi-natural experimental setting, use heavy-polluting and resource-based enterprises in pilot areas as the experimental group and heavy-polluting and resource-based enterprises in nonpilot areas as the control group, use Difference-in-Differences (DID) to test the influence of the OANRA on the innovation behaviors of those enterprises and discuss the role of government subsidies in this process.

This paper provides the following main contributions. First, this study examines the influence of changes to regional environmental regulations on the innovation behaviors of heavy-polluting and resource-based enterprises in pilot areas under an environment in which the Chinese government changes the assessment policies of local officials, which brings a new perspective for research on enterprise innovation. Current research on the factors that affect enterprise innovation behavior have mainly come from the perspective of corporate internal governance and external institutional backgrounds [4–6]. However, there are few studies that have been conducted from the perspective of the change assessment policies of local officials regarding the influence of the promotion incentive for officials on enterprise innovation behavior. This article expands the current research on enterprise innovation behavior. Second, this research supplements the existing studies of the OANRA. Previous empirical research has discussed the possible microeconomic consequences of OANRA pilots from the perspective of corporate earnings management and equity financing costs [7,8], while existing articles mainly focus on the meaning of the OANRA; relevant theoretical frameworks [9–11], modes [12], subjects, objects, contents and methods [13]; and its goals and implementation [14]. Therefore, this study further enriches the research in the field of the OANRA and expands on the possible microscopic consequences of the OANRA. Third, this article also expands on the study of the influences of local Chinese officials on microcosmic enterprise behaviors. Existing research has focused on changes of government officials, official inspections [15], and official corruption [16], while this article emphasizes the influence of the Chinese government’s changing assessment policies regarding local officials for enterprise behavior, which expands the existing research in this field.

The remainder of this paper is organized as follows. Section 2 consists of the institutional background, literature review and research hypothesis. Section 3 presents the research design. Section 4 evaluates the empirical results and analysis. Section 5 provides further analysis. The last section presents the research conclusions and suggestions.

## 2. Institutional background, literature review and research hypothesis

### 2.1 Institutional background

The Decision on Major Issues Concerning Comprehensively Deepening Reforms was adopted at the close of the Third Plenary Session of the 18^th^ Central Committee of the Communist Party of China in November 2013. The Decision issues a clarion call that to “Explore and establish a natural resources balance sheet, officials will receive audits on natural resources when leaving office. A lifelong responsibility system for bioenvironment damage will be established”. The original intention of the OANRA was to improve the assessment standards for leading cadres and reverse the current situation in which the leading cadres only pursue regional GDP growth and sacrifice natural resources and the ecological environment. The OANRA mainly focuses on monitoring whether leading officials obey environmental protection laws and regulations, comply with natural resource management and ecosystem protection targets, and perform their environmental supervision responsibilities. At the same time, the central government evaluates the officials’ performance related to natural resource management and ecosystem protection according to the audit results. Since 2014, more than 10 jurisdictions, including Shandong, Hubei, Inner Mongolia, Jiangsu, Fujian, have started the relevant pilot work of the OANRA. According to National Audit Office of the People's Republic of China [17], details of the pilots are shown in Table 1.

**Table 1:** OANRA pilots and content.

Province	Pilot City	Start time	Audit content
Shandong	Qingdao, Yantai	2014.3	Marine resource assets
Hubei	Huanggang	2014.4	List of natural resource assets
	Jiangxia District, Wuhan	2014.7	Economic responsibility audit and ecological environmental audit
Inner Mongolia	Ordos, Chifeng	2014.5	Protection of natural resources during the mayor’s term of office
Hunan	Loudi	2014.7	Responsibility for the development of mineral resources and protection of cultivated land and the environment
Guizhou	Chishui	2014.7	River Basin Natural Resources Audit
Jiangsu	Lianyungang	2014.6	State of marine resources and environmental protection of marine ecology
Fujian	Fuzhou	2014.7	Ecological protection of wetland environment and comprehensive improvement of water environment
	Wuyishan	2014.7	The particularity of Wuyishan World Natural Heritage and World Cultural Heritage
Guangxi	All cities	2014.11	Development and utilization of natural resource assets
Shanxi	Xi’an	2014.3	Natural resources balance sheet
Sichuan	Mianyang	2014	Ecological space and ecological environment
Guangdong	Bao’an District, Shenzhen	2014.8	Protection of natural resource assets

### 2.2 Literature review

At present, research on the relationship between government environmental protection and enterprise innovation mainly focuses on the perspectives of “complying with cost” and “innovation compensation”. From a static point of view, environmental protection is considered to bring high economic costs when technology levels, production processes, and consumption demands remain unchanged and to hinder the improvement of manufacturer productivity and international market competitiveness [18]. Environmental protection is viewed as not being conducive to technological innovation by enterprises, which is consistent with traditional neoclassical theory; although environmental protection can improve the overall welfare of society, it can reduce the technological innovation ability of enterprises by increasing the production costs of manufacturers. This increase leads to a reduction in the willingness and ability of enterprises to innovate technologically. Generally, more stringent environmental protection systems have higher requirements for enterprises. Palmer [19] found that stricter environmental regulations resulted in costs of environment protection that were higher than the profits generated by the new technology, which weakens the ability of companies to generate profits. Ramanathan [20] also believes that strict environmental regulation does not bring enough benefits for enterprises to cover their environmental protection costs and does not promote technological progress. The above results show that environmental protection policies can lead to a decline of enterprise performance and thus limit enterprise innovation, which supports the traditional view of “complying with cost”.

The “innovation compensation” concept discusses this problem from a dynamic perspective. Researchers have found that proper environmental protection regulations will stimulate technological innovation by enterprises. The innovative benefits can compensate for part or all of the production cost. This concept is the famous “Porter Hypothesis” [21]. Hamamoto [22] and Ambec [23] studied the relationship between environmental regulation and technological innovation and found that pollution control produced an incentive for R&D investment. Wagner [24] and Yang [25] believe that enhancing the strength of environmental protection regulations will ultimately promote enterprise profit growth by enterprise innovation, which can somewhat compensate for environmental management costs.

From the above research findings, we find that there is no consensus on whether environmental protection regulation policies can motivate enterprise innovation. The possible reason for this lack of consensus is that there are differences in the measurement standards for government environmental protection regulations. On one hand, many indicators have been adopted to measure environmental protection regulations, including GDP per capita [26] and pollutant emission intensity [27]. Measurement of environmental regulations based on these indicators is uncertain. On the other hand, government environmental protection regulation policies and enterprise innovation investments have serious endogenous problems. In this paper, based on the quasi-natural experimental environment of the OANRA from 2014, the influence of government environmental protection regulations on enterprise innovation can be alleviated to certain extent.

### 2.3 Research hypothesis

In the transitional economy, leading officers of local governments have a strong influence on enterprise behavior [3]. The local government controls large amounts of resources and has a very strong influence over resource configuration [1], which can affect resource acquisition by local enterprises [28] and influence local economic policies [2], which can further impact enterprise behavior in this area. Heavy-polluting and resource-based enterprises are the sources of natural resource consumption and ecological environmental pollution, which directly impact the improvement of natural resource environments in the pilot areas. Therefore, they will become the key regulatory objects of local government leaders [7]. Under the restraint of the accountability mechanism of the OANRA, local government officials will adopt strict environmental regulation measures for local heavy-polluting and resource-based enterprises, such as setting emission limits for waste gases and waste water produced during production or by requiring enterprises to adopt improved pollution control technologies to reduce pollution emissions, which will undoubtedly increase the cost of pollution control. Unlike existing environmental regulations, the OANRA assesses regional economic growth and the protection of natural resources assets within the terms of office of officials at the same time. Local officials need to not only achieve economic growth goals but also complete the task of environmental protection while improving energy efficiency through technological innovation as a key means of reducing pollution while protecting the environment and achieving sustainable development [29]. Therefore, local officials need to guide enterprises to implement technological innovations to protect the resource environment and grow the local economy. On the other hand, when facing the pressure of environmental protection from local governments, enterprises that pursue their own profit maximization usually adopt technological innovation to improve their pollution control capability in production processes and thereby decrease or counteract the environmental costs introduced by government environmental control, which is consistent with the “innovation compensation effect” [24,25]. As a result, we predict that the OANRA pilot areas will increase the investments by heavy-polluting and resource-based enterprises. Based on the above analysis, this paper proposes the following hypotheses.

H1: Compared with the heavy-polluting and resource-based enterprises in nonpilot areas, those companies in OANRA pilot areas invest more in innovation after the pilots.

However, the key to technological innovation relies on sufficient financial support. According to pecking order theory, enterprises usually prefer using internal sources to raise funds, while the OANRA requires enterprises to develop local economies and protect the ecological environment at the same time, which reduces available discretionary funds within the enterprise and leads to seeking more external financing. The long return period and uncertainty of innovation activities increase the difficulty of enterprise financing [30]. As a result, companies may reduce their investments in technological innovation due to low fund supplies. For this type of situation, government subsidies, as an important means of supporting enterprise innovation [31,32], can help to solve the shortage of enterprise innovation funds. On the one hand, government subsidies can provide financial support for enterprises in a direct or indirect manner and are a powerful external financing source for enterprise technological innovations, which reduces innovation investment costs and greatly motivates enterprises to innovate. Previous research has suggested that government subsidies are conducive to promoting enterprise investments in innovations [33,34]. On the other hand, government subsidies are a positive signal to external potential investors [35] and thus help to alleviate the problem of enterprise information asymmetry and improve the external financing capability. According to signaling theory, government subsidies provide signals that governments support and encourage industrial development, which brings crowding-in effects from external financing, reduces financing costs and increases investments in innovation [36]. Based on the above discussion, government subsidies can play a positive role in the process of the OANRA by impacting enterprise innovation investments. Based on the above analysis, this paper proposes the following assumption.

H2: Under the support of government subsidies, compared with heavy-polluting and resource-based enterprises in nonpilot areas, those companies in OANRA pilot areas have more investments in innovation after the pilot.

## 3. Research design

### 3.1 Sample selection and data sources

Since the OANRA pilot began in certain regions in 2014, this article selects all heavy-polluting and resource-based enterprises as samples from 2011 to 2016. Resource-based enterprises mainly include agriculture, forestry, animal husbandry, and fisheries, and heavy-polluting enterprises include the oil and gas industry, metal mining industry, textile industry, paper industry, petrochemical industry, metal smelting industry, and electric power production industry. According to the notice “Classification Management Catalogue of the Environmental Check Industry of Listed Companies” issued by the Ministry of Environmental Protection of the People’s Republic of China (the Ministry of Environmental Protection of the People’s Republic of China), this article defines the following industries as heavy-polluting industries: the coal mining and washing industry; the oil and gas mining industry; the black metal mining industry; the nonferrous metal mining industry; the textile industry; the leather, fur, feathers and products and footwear industry; the paper-making and paper products industry; oil processing; coking and nuclear fuel processing; chemical and chemical manufacturing, chemical fiber manufacturing; rubber and plastic products; nonmetallic mineral products; black metal smelting and pressure processing; nonferrous metal smelting and pressure processing; electricity; and the heat production and supply industry. According to the “Industry Classification of Listed Companies Guide”, which was revised by the China Securities Regulatory Commission in 2012, the codes for the heavy-polluting industries are B06, B07, B08, B09, C17, C19, C22, C25, C26, C28, C29, C30, C31, C32, and D44. We selected heavy-polluting and resource-based enterprises in the pilot areas in 2014 as the experimental group and those in nonpilot areas as the control group. We applied the DID model to compare the differences in innovation investment between the two groups. The pilot area information was obtained from the audit department website of each province: Shandong Province (Qingdao and Yantai), Hubei Province (Huanggang, Wuhan Jiangxia District), Inner Mongolia (Ordos and Chifeng), Hunan Province (Loudi), Guizhou Province (Chishui), Jiangsu Province (Lianyungang), Guangxi Province, Fujian Province (Fuzhou and Wuyi Mountain), Shaanxi Province (Xi’an), and Sichuan Province (Mianyang). We removed samples that containeds missing data. In the treatment and control groups, there were 2,579 observation samples over 6 years. A total of 286 samples belonged to the treatment group, and 2,293 were from the control group. Financial and corporate governance data were obtained from the CSMAR database. The standard errors of all regression models were processed by cluster at the corporate level.

### 3.2 Model and variable definition

The DID model is a widely used method in research that assesses policy effects. Flammer [37] used the DID model to study the impact of tariff reductions on corporate social responsibility. Dang et al. [38] examined the impact of anti-corruption policies on corporate innovation by a DID model. Yin et al. [39] adopted a DID model to study the impact of targeted measures in poverty alleviation on loans granted to farm households. Therefore, this paper chose the DID model to study the impact of the OANRA on enterprise innovation. As shown in Table 2, the basic concept of DID is to construct differences-in-differences statistics that reflect policy effects by comparing the differences between control groups and treatment groups before and after policy implementation.

**Table 2:** DID model principle.

	**Before policy**	**After policy**	**Difference**
**Treatment group**	α_0_+α_1_	α_0_+α_1_+α_2_+α_3_	α_2_+α_3_
Control group	α_0_	α_0_+α_2_	α_2_
Difference	α_1_	α_1_+α_3_	α_3_ (DID)

$$\begin{array}{l} RDS=\alpha_{0}+\alpha_{1}TREAT+\alpha_{2}POST+\alpha_{3}TREAT*POST+\alpha_{4}SIZE+\alpha_{5}LEV+\alpha_{6}ROA+\\ \alpha_{7}GROWTH+\alpha_{8}CFO+\alpha_{9}PPE+\alpha_{10}AGE+\alpha_{11}LOSS+\alpha_{12}INDP+\alpha_{13}SOE+YEAR+\\ IND+\varepsilon \end{array}$$ (1)

$$\begin{array}{l} RDS=\alpha_{0}+\alpha_{1}TREAT+\alpha_{2}POST+\alpha_{3}TREAT*POST+\alpha_{4}TREAT*POST*Subsidy+\\ \alpha_{5}Subsidy+\alpha_{6}SIZE+\alpha_{7}LEV+\alpha_{8}ROA+\alpha_{9}GROWTH+\alpha_{10}CFO+\alpha_{11}PPE+\alpha_{12}AGE+\\ \alpha_{13}LOSS+\alpha_{14}INDP+\alpha_{15}SOE+YEAR+IND+\varepsilon \end{array}$$ (2)

In model (1) and (2), RDS is the level of enterprise innovation investment. Regarding the measurement of enterprise innovation, current practice mainly uses the index of the number of patents and the ratio of research and development, the ratio of research and development investment and enterprise value. This paper studies the impact of the OANRA on enterprise innovation investments. The OANRA pilot started in 2014, which means a lack of a long-term observations, while the patent period from R&D is quite long. As a result, the number of enterprise patents is not used as an indicator. R&D investment includes capitalization and expense numbers, which are measured in currency and have more comparability. In addition, the audited revenues disclosed in financial reports are more reliable. Therefore, this article uses the proportion of R&D costs to operating revenues to measure the innovation costs [40,41]. TREAT is a virtual variable. If a sample is located in the pilot area, the value of TREAT equals 1. Otherwise, its value is 0. POST is a virtual variable. Before the pilot, its value is 0. Its value equals 1 after the pilot. Subsidies represent government subsidies. We expect that the coefficient of TREAT*POST*Subsidy is positive, which means that, when compared with the control group, the experimental group has invested more in technological innovation with the support of government subsidies after the pilot. The following variables are controlled: Company scale (SIZE), corporate liability asset ratio (LEV), corporate performance (ROA), company growth (GROWTH), corporate cash flow (CFO), asset intensity (PPE), listed time (AGE), company loss (LOSS), board independence (INDP), and equity property (SOE). In addition, we control the annual (YEAR) and industry (IND) fixed effect, which is a residual item *ε*. Table 3 shows the variable definitions.

**Table 3:** Variable definition.

Variable Name	Abbreviations	Definition
Explained variable	RDS	R&D expense/Operating revenue
Explanatory variable	TREAT	Virtual variable. For experiment group, the value is 1; otherwise, the value is 0.
	POST	Virtual variable. For samples after 2014, the value is 1; otherwise, the value is 0.
Adjusting Variables	Subsidy	Ln (1+ government subsidy)
Control variable	SIZE	Ln (asset)
	LEV	Total liabilities/Total assets
	ROA	Net profit/Asset
	GROWTH	Incremental changes of sales revenue in the current year compared with the previous year
	CFO	Net cash flow of the company’s operating activities/Total assets
	PPE	Fixed assets/Total assets
	AGE	Total number of years in which the company is listed
	LOSS	If the net profit of the company is less than 0, the value is 1. Otherwise, the value is 0.
	INDP	Number of in dependent directors of the company/Number of board members
	SOE	Equity nature of the company; for state-owned enterprises, the value is 1; otherwise, the value is 0.

## 4. Empirical analysis

### 4.1 Descriptive statistics of the main variables

Table 4 shows the descriptive statistics of the main variables and univariate analysis. As shown in Panel AA, the average RDS is 0.0190, and the median is 0.0155, which indicates that the listed company investment in R&D is low. The average TREAT is 0.1109, which means that the number of samples located in the pilot area accounts for 11.09%. The average POST is 0.5130, which indicates that the proportion of samples before and after the pilot were relatively the same. The descriptive statistics of other variables were similar as in the previous research. Panel B shows the univariate analysis grouped by TREAT. With the exception of SIZE, ROA, AGE, and SOE, the difference tests of the variables were not significant, which illustrates that there were minor differences between the companies in the pilot and nonpilot areas. It indicates that the pairing of our control group and experimental group was valid. Panel C shows a univariate analysis grouped by POST. It can be seen that enterprise R&D investments significantly increased after OANRA implementation in pilot areas. In addition, other differences of control variables between groups are significant. It is necessary to adopt PSM to reduce the differences between groups.

**Table 4:** Description of the main variables and univariate analysis.

Panel A Descriptive statistical analysis								
Variable	Sample Size	Average value	Standard deviation	Upper quartile			Median	Lower quartile
RDS	2579	0.0190	0.0182	0.001			0.0155	0.0324
TREAT	2579	0.1109	0.3141	0			0	0
POST	2579	0.5130	0.4999	0			1	1
Subsidy	2579	16.7983	1.7833	15.8511			16.9303	17.8580
SIZE	2579	22.0335	1.2000	21.1641			21.8722	22.7027
LEV	2579	0.4756	0.2290	0.2954			0.4768	0.6499
ROA	2579	0.0241	0.0611	0.0060			0.0254	0.0547
GROWTH	2579	0.0835	0.1907	-0.0103			0.0672	0.1660
CFO	2579	0.0543	0.0850	0.0093			0.0541	0.0985
PPE	2579	0.3473	0.1758	0.2157			0.3284	0.4682
AGE	2579	10.4180	6.0870	5			11	16
LOSS	2579	0.1501	0.3572	0			0	0
INDP	2579	0.3694	0.0521	0.3333			0.3333	0.4
SOE	2579	0.4637	0.4988	0			0	1
Panel B Univariate analysis grouped by TREAT								
	TREAT = 0		TREAT = 1			Difference test		
variables	Obs.	Mean	Median	Obs.	Mean	Median	t-test	Median test
RDS	2293	0.019	0.015	286	0.02	0.018	-0.002	1.29
Subsidy	2293	16.804	16.931	286	16.75	16.928	0.055	0.014
SIZE	2293	22.058	21.911	286	21.837	21.666	0.221***	15.063***
LEV	2293	0.475	0.476	286	0.477	0.495	-0.001	0.147
ROA	2293	0.025	0.026	286	0.02	0.021	0.004	3.513*
GROWTH	2293	0.084	0.068	286	0.081	0.062	0.003	0.385
CFO	2293	0.054	0.054	286	0.054	0.051	0.001	0.385
PPE	2293	0.349	0.329	286	0.331	0.324	0.018	0.245
AGE	2293	10.489	11	286	9.696	9	0.793**	7.925***
LOSS	2293	0.148	0	286	0.168	0	-0.02	0.797
INDP	2293	0.369	0.333	286	0.373	0.333	-0.004	0.995
SOE	2293	0.451	0	286	0.566	1	-0.115***	13.640***
Panel C Univariate analysis grouped by POST								
	POST = 0		POST = 1			Difference test		
variables	Obs.	Mean	Median	Obs.	Mean	Median	t-test	Median test
RDS	1256	0.017	0.013	1323	0.021	0.018	-0.003***	7.500***
Subsidy	1256	16.646	16.799	1323	16.943	17.012	-0.297***	10.825***
SIZE	1256	21.886	21.741	1323	22.173	21.991	-0.287***	27.666***
LEV	1256	0.488	0.5	1323	0.464	0.455	0.025***	13.856***
ROA	1256	0.024	0.025	1323	0.024	0.025	-0.001	0.031
GROWTH	1256	0.083	0.076	1323	0.084	0.06	0	4.608**
CFO	1256	0.044	0.047	1323	0.064	0.061	-0.020***	25.235***
PPE	1256	0.343	0.321	1323	0.351	0.332	-0.009	1.175
AGE	1256	9.299	10	1323	11.447	12	-2.147***	17.672***
LOSS	1256	0.148	0	1323	0.152	0	-0.004	0.074
INDP	1256	0.368	0.333	1323	0.371	0.333	-0.003	0.941
SOE	1256	0.488	0	1323	0.441	0	0.047**	5.819**

### 4.2 Multiple regression analysis

Table 5 shows the regression results of model (1) and model (2). Column (1) in the table shows that the coefficient of the interactive TREAT*POST is not significant and indicates that the OANRA did not promote innovation activities by the companies in the region. Therefore, hypothesis H1 is rejected. However, environmental protection becomes a major appraisal measurement for local officials according to the OANRA. Local officials should focus on natural resource protection and protecting the ecological environment and strengthen the supervision of heavy-polluting and resource-based enterprises. However, from the enterprise perspective, local officials have made optimal choices under the same production technologies, resource allocations, and consumption requirements. Environmental protection activity only weakens production efficiency, international competitiveness [42–44] and innovation capability. These results are consistent with traditional neoclassical theory.

**Table 5:** The OANRA, government subsidies and enterprise innovation.

	(1)	(2)
Variable	RDS	RDS
TREAT	0.0013	0.0012
	(0.63)	(0.60)
Post	0.0106***	0.0100***
	(10.02)	(9.58)
TREAT*POST	-0.0017	-0.0360*
	(-1.06)	(-1.96)
TREAT*POST*Subsidy		0.0020*
		(1.87)
Subsidy		0.0014***
		(4.78)
SIZE	-0.0008	-0.0021***
	(-1.52)	(-3.76)
LEV	-0.0176***	-0.0185***
	(-6.56)	(-6.89)
ROA	-0.0005	-0.0028
	(-0.06)	(-0.33)
GROWTH	0.0053***	0.0056***
	(2.80)	(3.03)
CFO	0.0038	0.0040
	(0.87)	(0.95)
PPE	-0.0017	-0.0021
	(-0.49)	(-0.60)
AGE	-0.0011***	-0.0010***
	(-9.08)	(-8.80)
LOSS	-0.0007	-0.0003
	(-0.64)	(-0.27)
INDP	-0.0038	-0.0013
	(-0.44)	(-0.15)
SOE	0.0006	-0.0001
	(0.41)	(-0.05)
Constant	0.0374***	0.0415***
	(3.26)	(3.67)
Industry fixed effect	Controlled	Controlled
Annual fixed effect	Controlled	Controlled
Observations	2,579	2,579
R-squared	0.412	0.427

In the parentheses, t is the test value.

***, **, and * indicate significance levels of 1%, 5%, and 10%, respectively.

However, we can see that the TREAT*POST*Subsidy coefficient was significantly positive at 10% when government subsidies were used as the adjustment variable. This result shows that, compared, c with the nonpilot regions, with the support of government subsidies, the innovation investment for the pilot region significantly improved after the pilot, which supports hypothesis H2. The above results show that enterprises in the pilot area are faced with dual pressures to promote economic growth and protect the resource environment. The regions lack sufficient financial resources to invest in innovative activities with long terms and high risks. Government subsidies provide strong financial support to alleviate financial pressure on enterprises and encourage enterprise innovation. In terms of the economic significance, compared with companies in nonpilot areas, innovation investments of companies in the pilot regions have significantly improved by 10.52% (0.0020/0.0190) after the pilot. In the control variables, taking column (2) as an example, the company scales, liability asset ratios, and listed times were significantly negatively related to enterprise innovation because larger scale and more mature enterprises will reduce their innovation investments due to their influence in the market and will be satisfied with their current success and lack motivation to invest in innovation. Companies with higher liability asset ratios face greater financing limitations and therefore lack sufficient resources to invest in innovation. Government subsidies and corporate growth are positively correlated with enterprise innovation. These results indicate that government subsidies provide financial resources for enterprises to invest in innovation. Companies undergoing rapid growth exhibit a very high demand for innovations, sufficient cash flow, motivation and the capability to perform innovation activities.

### 4.3 Robustness test

To ensure the robustness of the above main test results, we performed the following robustness test.

First, to alleviate endogenous problems caused by missing variables, this paper used the propensity score matching method. We placed a sample that had similar company characteristics in terms of company size, liability asset ratio, book value to market value ratio, equity yield, and growth rate but was not located in a pilot region in the experimental group. Table 6 Panel A shows the regression result. The value of TREAT*POST*Subsidy was significantly positive. The results show that the research conclusion is still correct after excluding company characteristics and selective bias.

**Table 6:** Robustness test.

Panel A: PSM Test		
Variable	(1) RDS	(2) RDS
TREAT	0.0002	0.0001
	(-0.09)	(-0.04)
Post	0.0078***	0.0079***
	(-3.29)	(-3.38)
TREAT*POST	-0.0334**	-0.0021
	(-2.14)	(-0.94)
TREAT*POST*Subsidy	0.0018**	
	(-2.00)	
Subsidy	0.0008	
	(-1.42)	
Control variable	Controlled	Controlled
Industry fixed effect	Controlled	Controlled
Annual fixed effect	Controlled	Controlled
Observations	552	558
R-squared	0.468	0.462
Panel B: Balance Check		
Variable	(1) RDS	(2) RDS
TREAT	0.0018	0.0019
	(-0.81)	(-0.85)
Post	0.0111***	0.0117***
	(-9.56)	(-9.96)
TREAT*POST	-0.0374**	-0.0011
	(-2.00)	(-0.75)
TREAT*POST*Subsidy	0.0021*	
	(-1.91)	
Subsidy	0.0010***	
	(-3.1)	
Control variable	Controlled	Controlled
Industry fixed effect	Controlled	Controlled
Annual fixed effect	Controlled	Controlled
Observations	2,171	2,167
R-squared	0.441	0.436
Panel C: Deleted samples from Guangxi and Mianyang.	Mianyang	
Variable	RDS	RDS
TREAT	0.0033	0.0034
	(-1.3)	(-1.35)
Post	0.0100***	0.0065***
	(-9.36)	(-7.15)
TREAT*POST	-0.0541**	-0.0024
	(-2.48)	(-1.09)
TREAT*POST*Subsidy	0.0031**	
	(-2.4)	
Subsidy	0.0015***	
	(-5.02)	
Control variable	Controlled	Controlled
Industry fixed effect	Controlled	Controlled
Annual fixed effect	Controlled	Controlled
Observations	2,454	2,451
R-squared	0.428	0.415

In the parentheses, t is the check value.

***, **, and * indicate significance levels of 1%, 5%, and 10%, respectively.

Second, the sample companies before and after the pilot may not be completely consistent, and these differences may affect the conclusion. We retained the companies that existed before and after the pilot as a research sample and used the balanced panel data with six years of observations for analysis. Table 6 Panel B shows the regression result, and it can be seen that the research conclusion did not change.

In addition, Guangxi Province has had the OANRA implemented since November 2014. Since the pilot start time was close to the end of the financial year, local companies may not have been able to make accounting adjustments in time. The samples from Mianyang, Sichuan Province did not disclose the specific pilot start time in 2014. As a result, we removed samples from Guangxi Province and Mianyang City, Sichuan Province. Table 6 Panel C shows that the regression result and TREAT*POST*Subsidy was still significantly positive and thus indicated that our research conclusion did not change.

## 5. Further analysis

### 5.1 Company scale

In small-scale enterprises, the enterprise spirit can be more effective than it is in large-scale companies. Therefore, small enterprises are more flexible and efficient in innovation. Highly specialized and competitive small-scale companies are the basic unit of industry upgrades [45]. However, small enterprises face greater financing constraints since enterprise scales are positively related with financing. Enterprise innovation activities require large amounts of R&D funding, while the long return period and uncertainty of innovation activities increase the difficulty of obtaining enterprise financing [30]. From the above analysis, financing constraints become the “bottleneck” for the innovation activities of small enterprises. The OANRA brings pressure on local enterprises to protect the local environment, but enterprises of different scales have different abilities to resist such pressure. Large-scale enterprises have strong financial support and are capable of promoting environmental protection through innovation, but the incremental effect of the OANRA is smaller than it is for small-scale companies. Small-scale enterprises cannot engage in more innovative activities due to their limited capabilities. With government subsidies, they could increase investment in innovation activities to promote environmental protection and this would relieve pressure from government officials. Therefore, we predict that this effect would be reflected in small-scale companies with innovative capability but lack financial support under the support of government subsidies. To test the above assumptions, we divided the samples into groups of large companies and small companies based on the median value of the company scale variable. Table 7 (1) shows the regression result. It shows that TREAT*POST*Subsidy is significant for small-scale companies but is not significant for large-scale companies which is consistent with our predictions.

**Table 7:** Further analysis.

	(1)		(2)		(3)		(4)	
Variable	Large scale	Small scale	State-owned enterprises	Non-state-owned enterprises	Good	Poor	High credit constraints	Low credit constraints
TREAT	0.0065***	-0.0013	0.0050*	-0.0036	-0.0012	0.0022	0.0017	0.0016
	(3.31)	(-0.80)	(1.78)	(-1.18)	(-0.42)	(0.94)	(0.59)	(0.57)
Post	0.0066***	0.0106***	0.0093***	0.0119***	0.0088***	0.0064***	0.0056***	0.0067***
	(4.95)	(7.00)	(5.80)	(7.46)	(6.41)	(4.49)	(4.64)	(4.92)
TREAT*POST	-0.0250	-0.0334*	-0.0386*	-0.0252	-0.0710***	-0.0151	-0.0533*	-0.0237
	(-1.15)	(-1.83)	(-1.75)	(-0.91)	(-2.93)	(-0.71)	(-1.80)	(-1.15)
TREAT*POST*Subsidy	0.0012	0.0019*	0.0022*	0.0015	0.0042***	0.0008	0.0030*	0.0013
	(1.01)	(1.72)	(1.66)	(0.87)	(3.01)	(0.64)	(1.70)	(1.02)
Subsidy	0.0011***	0.0015***	0.0006*	0.0012***	0.0015***	0.0013***	0.0009**	0.0020***
	(4.14)	(5.15)	(1.96)	(3.99)	(3.43)	(3.63)	(2.49)	(4.77)
Control variable	Control	Control	Control	Control	Control	Control	Control	Control
Industry fixed effect	Control	Control	Control	Control	Control	Control	Control	Control
Annual fixed effect	Control	Control	Control	Control	Control	Control	Control	Control
Observations	1,290	1,289	1,196	1,383	1,307	1,272	1,361	1,218
R-squared	0.342	0.450	0.332	0.303	0.437	0.374	0.390	0.457

In the parentheses, t is the check value.

***, **, and * indicate significant levels of 1%, 5%, and 10%, respectively.

### 5.2 Company ownership

Local officials face the pressures of environmental protection and economic development due to the OANRA requirements. State-owned enterprises in China have inherent resource endowments, while private enterprises often face serious credit discrimination and financing constraints [46]. The differences in property types not only bring differences in resource acquisition ability and innovation ability but also influence the effects of government subsidies. Compared with non-state-owned enterprises, state-owned enterprises have the following characteristics: large scales, strong investment capabilities, low intervention costs, and government relationships [47]. Therefore, local officials are more likely to grant funds to state-owned enterprises when facing the OANRA to ensure that natural resources and the ecological environment are protected while promoting economic development in a region. To test the above results, we classified the research samples into groups of state-owned and non-state-owned enterprises according to the property type of the enterprise. Table 7 (2) shows the regression result. TREAT*POST*Subsidy is significant in the state-owned enterprise group but not for private enterprises. This suggests that the positive effects of the pilot on enterprise innovation investment is more likely to occur for state-owned enterprises with the support of government subsidies. Compared with non-state-owned enterprises, state-owned enterprises can obtain more government subsidies, which further supports adjustment of government subsidies to the OANRA and enterprise innovation.

### 5.3 Institutional environment

The institutional environment affects the appraisal and promotion of local government officials [7]. In areas with good institutional environments, execution by the institution is strong, and officials are constrained and supervised by the OANRA. The economic development level is high and acts as a solid economic foundation for protecting natural resources and the ecological environment. If the OANRA promotes the innovation investment of local enterprises by changing the appraisal methods of local officials, we propose that this positive effect should be more significant in areas with good institutional environments. Therefore, according to the median value of the marketization index of the region of the company’s location, the research samples were divided into two groups: good institutional environments and poor institutional environments. This paper measures marketization according to China’s provincial marketization index report [48]. Table 7 (3) shows the regression test result. In the group with a good system environment, TREAT*POST*Subsidy is significantly positive at a 1% level, which indicates that under the financial support of the government, the OANRA promotes innovation investment by relevant enterprises in these areas. This support is mainly due to the good institutional environments providing an effective guarantee for the OANRA in protecting natural resources and the ecological environment. In the group with poor institutional environments, the TREAT*POST*Subsidy is not significant, which indicates that the OANRA has no impact on the innovation investments of relevant enterprises in areas with poor institutional environments. This lack of impact is because the OANRA is not able to constrain the behavior of officials in these areas, and officials lack the motivation to supervise local enterprises. According to the above results, the influence of the OANRA on the innovation investments of the relevant companies only exists in those areas with good institutional environments.

### 5.4 Financing constraints

Bank credit plays a leading role in Chinese financial activities. As the cost of debt financing is lower than that of equity financing, bank loans are the major financing resource for enterprises. However, research shows that bank credit constraints affect innovation investments by companies. There is a principal-agent problem between banks and corporate management [49]. Banks use contingent governance to restrict innovation projects to curb overinvestment; therefore, enterprises may reduce their innovation investments [50]. The conflict between the fixed nature of bank debt repayments and the fluctuating cash flow of innovation projects causes enterprises with higher credit strengths to face strong liquidity constraints, which thus further restricts innovation investment. When bank credit highly constrains enterprises, the possibility of enterprises involving innovation investments is significantly reduced, while government subsidies can fill the financing gap. If enterprises in pilot areas are engaged in more innovative activities with government subsidies, we assume that this effect is more significant for enterprises with higher bank credit constraints. This paper uses the proportion of company bank loans to corporate assets to measure the level of company bank credit constraints. We tested model (2) according to the median number of bank credit constraints. Table 7 (4) shows the regression result. In companies with higher bank credit constraints, TREAT*POST*Subsidy is significantly positive at 10%, which suggests that under the financial support of the government, the OANRA makes more significant contributions to innovation investments by companies when facing large bank credit constraints.

## 6. Conclusions

Since the reform and opening up, local Chinese governments have pursued GDP targets but have neglected environmental protection due to the lack of environmental monitoring mechanisms. In this situation, Chinese governments implemented the OANRA in pilot areas so that officials’ appraisals are determined by economic growth and ecological protection. The OANRA may impact supervision behavior on heavy-polluting and resource-based enterprises and then affect the technologic innovation of these companies. By exploring the relationship between the OANRA and the innovation investments of enterprises, this paper suggests that the OANRA cannot significantly affect the technological innovation behavior of enterprises. Considering the adjustment effect of government subsidies, this paper empirically tested the relationship between the OANRA and the innovation investments of heavy-polluting and resource-based enterprises. The results show that, with the support of government subsidies, the impact of the OANRA on the innovation investments of heavy-polluting and resource-based enterprises in the pilot areas becomes significant. The more government subsidies that these companies receive, the more they can stimulate their innovation investments.

In addition, we also found that, with the support of government subsidies, enterprises with small-scale, state-owned, high bank credit and in high marketization regions significantly promoted enterprise innovation investment after OANRA implementation. At present, China is in a critical stage of economic development and is transforming to a new normal state. It is important to continuously improve the innovation capability of enterprises and promote the implementation of innovative development strategies to achieve economic transformation and upgrades. According to the research findings in this article, we propose the following policy suggestions. (1) This paper finds that the OANRA is not able to directly promote the innovation activities of enterprises. The current audit system still needs improvement. Therefore, to effectively protect natural resources, we must establish and improve the laws and regulations on natural resource asset management and further clarify the responsibility of local officials to protect natural resources. (2) This article finds that government subsidies, as an effective financial tool, can drive enterprise innovation investments in the pilot areas. Therefore, it is necessary for the government to increase government subsidies for enterprises in pilot areas while implementing the OANRA.

The limitations of this paper are the time span of the samples and data errors. First, due to data acquisition limitations, this paper used the policy pilot before and after 3 years as the research interval, but a comprehensive objective assessment of the effects of the policy needs to examine longer times and a wider range of resources. Second, in China, most government subsidies that were granted to listed companies were nonmonetary asset projects, which make such subsidies difficult to be reflected in financial reports. Therefore, there may be errors in the government subsidy data in this paper.

With the further publication of relevant data, future research can be further carried out in the following areas: (1) expand the research scope to longer periods and then observe whether the findings of this paper are still valid; (2) after national implementation of the OANRA, the pollution and nonpolluting industries can be defined as the treatment group and control group, respectively, to examine the impact of the policy on enterprise innovation; and (3) further examine the impact of the OANRA on company environmental protection investments and other aspects.

## Supporting information

S1 File (RAR)
