# Correction: The effect of environmental regulations on innovation in heavy-polluting and resource-based enterprises: Quasi-natural experimental evidence from China

**DOI:** 10.1371/journal.pone.0272098

**Published:** 2022-07-21

**Authors:** The PLOS Journal Staff

The affiliation for the second author is incorrect. The correct affiliation for Meijuan Liu is: School of Economics and Management, Zhejiang A&F University, Hangzhou, China.

The affiliation for the third author is incorrect. The correct affiliation for Xiaohan Hu is: Faculty of Agriculture and Forestry, University of Helsinki, Finland.

The publisher apologizes for the errors.
